# Highlighting the Need for MD-PhD Programs in Developing Countries

**DOI:** 10.1055/s-0043-1768445

**Published:** 2023-04-26

**Authors:** Laith Ashour, Ali Schoumann-Alkhatib, Anmar Alshawabkeh, Mohammad Alsouri, Mohammad Sawalmeh, Hamzeh Hatamleh, Hamza Sawahreh

**Affiliations:** 1 Faculty of Medicine, Al-Balqa Applied University, Al-Salt, Jordan

In certain developing countries, research output in the medical field has been rising rapidly in the last few decades due to an increased focus on medical research development through different avenues. 1 These indicated an increase in the expenses on research projects, the launch of more research centers, and others. 1 2 However, this is not the case for most developing countries. Compared with developing countries, developed countries have contributed a lot more to scientific publications in health research. 3 4 Furthermore, the health research productivity of postgraduate students in low- and middle-income countries was found to be low. 5 In detail, the majority of students infrequently publish their theses. In addition, most published studies are cross-sectional in design, with hardly any clinical trials. 5 The reasons behind that vary, but many of them are common, including limited funding and resources, weak linkage between universities and stakeholders, limited facilities for research training and education, and limited access to health information and literature sources. 3 6 Such problems have affected medical education in developing countries negatively. For example, in one of the low-income countries, Mozambique, for the period between 2004 and 2010, only 11% of Mozambican University faculty members had a PhD degree, and the shortage of human resources for teaching and mentoring in that country is still an ongoing problem. 7 It has also become clear that developing nations' clinical research needs to be improved if important issues are to be resolved and effective pandemic management tactics are to be selected. As an illustration, the coronavirus disease 2019 (COVID-19) pandemic, which started in the Chinese city of Wuhan in 2019 and is brought on by the severe acute respiratory syndrome coronavirus 2 virus that mostly affects the respiratory tract, 8 has had a major impact on public health in India. Following the second wave of COVID-19 in India, it has been argued that conducting research is essential for low- and middle-income countries so that resources can be allocated properly where they are needed and health systems can develop a greater understanding of what causes good and bad outcomes. 9

In light of these facts, we highlight an important, underestimated, and poorly applied approach for the improvement in medical research and education in developing countries, that is, the implementation of Doctor of Medicine-Doctor of Philosophy (MD-PhD) programs.

## What Is the MD-PhD Program?

The MD-PhD program is a special program that provides a course that integrates the rigor of an MD program in science and medicine with the rigor of a PhD program in science and research, allowing physician-scientists to apply clinical medicine knowledge to a wide range of biomedical research. 10 Preclinical (basic medical sciences), clinical, and PhD research phases make up the three phases of the dual degree. 11 The dual-degree program's prerequisites are typically completed by students in 8 years. 11 In contrast, the MD program lasts only 4 years in the United States, where medical students are regarded as graduate students. Another situation involves an accelerated BS-MD curriculum that enables students to complete both their undergraduate and medical degrees in 6 years. 12 However, in other nations (such as Jordan), the MD program is a 6-year bachelor's degree program with undergraduate students as its participants.

## MD-PhD Programs and Research Output

The MD-PhD program was introduced in the United States in 1956 and is now performed in different countries, with the majority being developed ones, like the United Kingdom, the Netherlands, Germany, Switzerland, and others. 13 The impact of such programs on the research output of developed countries was and is still significant. For example, in the United States, although MD-PhD graduates comprise a small proportion of all physicians, they make a unique contribution to the medical field. 14 Survey research evaluating 24 MD-PhD programs in the United States found that 81% of the graduates were employed in academia, research institutes, or industry, and of those in academia, the majority (more than 80%) did research and 61% had identifiable funding. 15 This demonstrates the crucial role MD-PhD candidates play in raising the level of medical research on a national basis. We read these results while bearing in mind that, with more than 4,900,000 documents published only in 2021, the United States is at the top of the Scimago country rankings for the subject “Medicine.” 16 In another example, in Canada, a survey study found that the MD-PhD students at the University of Ottawa engage heavily in scholarly activities, with an average of 8.3 presentations or publications per student. 17 Away from specification, it was found that more than half of the Nobel Prize in Physiology or Medicine laureates in the period between 1997 and 2013 were physician-scientists or teams with at least one physician-scientist. 10

The enormous impact that MD-PhD programs have on research output is highlighted by these findings, which promote the development of similar programs in developing countries to advance medical research and productivity there.

## Is It Feasible to Implement MD-PhD Programs in Developing Countries?

Someone may ask, Are the incentives needed to conduct an MD-PhD present among medical students in developing countries? Well, the answer is simple. It was found that students enroll in the program with plans to continue in a research-oriented career, such as in industries, biomedical research, research institutes, or a career that combines medical patient care with research. 18 19 In other words, they intend to start an MD-PhD because of their interest in conducting research.

Although different factors were found to potentially affect medical students' attitudes toward research negatively, such as the lack of time given competing educational demands, the timing of family for married students, low prior awareness of research principles, and others, studies of medical students in developing countries found them to have the interest to pursue research. 20 By reviewing the studies examining research interest among medical students in developing nations, we discovered that while participation rates are low, research interest is high in many developing countries, including Brazil, Jordan, Egypt, India, and others. 21 22 23 24 25 Several of the causes of that have already been discussed, 3 6 and the introduction of MD-PhD programs would be a potential opportunity for medical students to engage in research in fields of interest.

## The Optimal Implementation of MD-PhD Programs in Developing Countries

To ensure the best implementation of MD-PhD programs, high schools and colleges should network with MD-PhD programs' students, directors, and mentors to help students learn about MD-PhD programs and careers as physician-scientists and to allow MD-PhD program' students to collaborate in research with MD-PhD programs in developed countries, which would aid in providing funding for developing countries' students. 26 27 In addition, we can encourage MD-PhD students to network with other researchers, encourage them to attend scientific conferences, and emphasize research as a learning process and reduce focus on output. 28 Furthermore, we can enhance MD-PhD students' engagement in clinical research by providing clinical research experiences during training to develop the self-efficacy of students' research skills. 29 We must also prevent the process of “brain drain” affecting skilled medical students in developing countries from catching up with the graduates of MD-PhD programs. 30 31 This can be achieved by providing a clear career path for the graduates, expanding programs for faculty development, supporting health professions education scholarship units, and making criteria for academic advancement and professorship dependent on the research output of these graduates, which motivates them to stay in their countries due to the availability of the necessary support and motivation to complete their research projects. 32 33 Of course, we should focus on providing adequate funding for the programs and research projects through adequate collaboration between universities and local or international governments and/or investors.

## Concluding Remarks

MD-PhD programs are powerful educational programs to advance medical education and research in developing countries. Although many papers have addressed different ways of enabling research in such countries, MD-PhD programs were poorly discussed in the literature as a solution to the low research outcomes seen in developing countries. We have discussed the need for these programs in developing countries, the feasibility of such programs for medical students there, and recommendations for the best implementation of these programs in developing countries. The main strategies for successful program implementation include allowing MD-PhD program students to collaborate in research with MD-PhD programs in developed countries (which would help provide funding for students in developing countries), ensuring adequate funding for programs and research projects through effective cooperation between universities and local or international governments and/or investors, preventing the process of “brain drain” that negatively affects developing countries, and empowering linkage between universities and stakeholders.
